# Late effects of pediatric hematopoietic stem cell transplantation on left ventricular function, aortic stiffness and myocardial tissue characteristics

**DOI:** 10.1186/s12968-018-0513-4

**Published:** 2019-01-17

**Authors:** Elisabeth H. M. Paiman, Marloes Louwerens, Dorine Bresters, Jos J. M. Westenberg, Qian Tao, Rob J. van der Geest, Arjan C. Lankester, Arno A. W. Roest, Hildo J. Lamb

**Affiliations:** 1 Department of Radiology, Leiden University Medical Center, P.O. Box 9600, postal zone C2-S, 2300 RC Leiden, The Netherlands; 2 Department of Internal Medicine, Leiden University Medical Center, P.O. Box 9600, postal zone C7-Q, 2300 RC Leiden, The Netherlands; 3 Department of Pediatrics, Leiden University Medical Center, Leiden, The Netherlands; 4 Princess Máxima Center for Pediatric Oncology, Utrecht, The Netherlands

**Keywords:** Hematopoietic stem cell transplantation, Pediatric, Cardiovascular magnetic resonance, Systolic and diastolic function, Aortic stiffness, Diffuse fibrosis, Myocardial steatosis, T1 mapping

## Abstract

**Background:**

Pediatric hematopoietic stem cell transplantation (HSCT) recipients are at increased risk of cardiovascular disease later in life. As HSCT survival has significantly improved, with a growing number of HSCT indications, tailored screening strategies for HSCT-related late effects are warranted. Little is known regarding the value of cardiovascular magnetic resonance (CMR) for early identification of high-risk patients after HSCT, before symptomatic cardiovascular disease manifests. This study aimed to assess CMR-derived left ventricular (LV) systolic and diastolic function, aortic stiffness and myocardial tissue characteristics in young adults who received HSCT during childhood.

**Methods:**

Sixteen patients (22.1 ± 1.5 years) treated with HSCT during childhood and 16 healthy controls (22.1 ± 1.8 years) underwent 3 T CMR. LV systolic and diastolic function were measured as LV ejection fraction (LVEF), the ratio of transmitral early and late peak filling rate (E/A), the estimated LV filling pressure (E/Ea) and global longitudinal and circumferential systolic strain and diastolic strain rates, using balanced steady-state free precession cine CMR and 2D velocity-encoded CMR over the mitral valve. Aortic stiffness, myocardial fibrosis and steatosis were assessed with 2D velocity-encoded CMR, native T1 mapping and proton CMR spectroscopy (^1^H-CMRS), respectively.

**Results:**

In the patient compared to the control group, E/Ea (9.92 ± 3.42 vs. 7.24 ± 2.29, *P =* 0.004) was higher, LVEF (54 ± 6% vs. 58 ± 5%, *P* = 0.055) and global longitudinal strain (GLS) ( -20.7 ± 3.5% vs. -22.9 ± 3.0%, *P =* 0.063) tended to be lower, while aortic pulse wave velocity (4.40 ± 0.26 vs. 4.29 ± 0.29 m/s, *P =* 0.29), native T1 (1211 ± 36 vs. 1227 ± 28 ms, *P =* 0.16) and myocardial triglyceride content (0.47 ± 0.18 vs. 0.50 ± 0.13%, *P =* 0.202) were comparable. There were no differences between patients and controls in E/A (2.76 ± 0.92 vs. 2.97 ± 0.91, *P =* 0.60) and diastolic strain rates.

**Conclusion:**

In young adults who received HSCT during childhood, LV diastolic function was decreased (higher estimated LV filling pressure) and LV systolic function (LVEF and GLS) tended to be reduced as compared to healthy controls, whereas no concomitant differences were found in aortic stiffness and myocardial tissue characteristics. When using CMR, assessment of LV diastolic function in particular is important for early detection of patients at risk of HSCT-related cardiovascular disease, which may warrant closer surveillance.

## Background

Hematopoietic stem cell transplantation (HSCT) recipients are exposed to several pre-transplant and/or HSCT-related therapies which may increase the risk of cardiovascular disease [1, 2]. As HSCT survival has significantly improved over the last decades [3, 4], with an increasing number of HSCT indications for both malignant and non-malignant disease [5], targeted follow-up strategies for the HSCT population are needed [6]. Recently, several international working groups have been established aimed at a greater understanding of the late effects including arterial disease and cardiac dysfunction [7, 8]. Most of the available studies on late effects involve HSCT in adults. Adequate screening in young HSCT recipients is even more challenging [9].

According to current guidelines [10], pediatric HSCT recipients who have an increased susceptibility to complications later in life, based on pre-existing comorbidities, pre-transplant exposures, the HSCT preparative regimen, post-transplant complications such as graft-versus-host-disease, or relapse of the primary disease, are selected for patient-specific follow-up programs. Identification of imaging markers which indicate subclinical disease would be supportive in the detection of high-risk patient groups for closer monitoring or targeted therapy [6]. In general, clinical follow-up after cardiotoxic exposures comprises echocardiography for left ventricular (LV) systolic and diastolic function. Less is known regarding the value of cardiovascular magnetic resonance (CMR) for screening of late effects [11]. CMR may be suited for comprehensive evaluation of subclinical deteriorations within the cardiovascular system after HSCT, that may be present before overt LV functional abnormalities arise.

Pre-transplant or HSCT-related cardiotoxic exposures may cause endothelial damage leading to increased aortic stiffness and myocyte cell death with reactive interstitial fibrosis [1, 2]. In addition, the immunosuppressive therapies in allogeneic HSCT increase the susceptibility to developing the metabolic syndrome at young age [12]. Aortic stiffening is known to occur in relation to normal, physiological ageing [13], but will be more progressive in response to hypertension, dyslipidemia or hyperglycemia [14] and possibly due to iron overload [15]. Increased aortic stiffness induces LV concentric remodeling and is recognized as an independent predictor for cardiovascular events [16]. Myocardial diffuse fibrosis in different types of cardiomyopathies is considered to reflect subclinical disease before cardiac dysfunction becomes manifest [17]. The metabolic derangements among allogeneic HSCT recipients may predispose to myocardial steatosis. In individuals with the metabolic syndrome, myocardial steatosis has been associated with LV remodeling [18].

We hypothesize that, when using CMR, subclinical deteriorations in LV function, aortic stiffness and/or myocardial tissue characteristics can be detected in young adults who received HSCT during childhood. Therefore, CMR may be suitable for early identification of patients at increased risk of developing HSCT-related cardiovascular disease. Accordingly, the aim of this study is to assess CMR-derived LV systolic and diastolic function, aortic stiffness and myocardial fibrosis and steatosis in young adults who have received pediatric HSCT and to compare these measures with those in healthy controls in the same age range.

## Methods

### Study population

The patient group consisted of young adults (18 to 25 years old), who received HSCT for malignant or non-malignant disease during childhood. Patients were recruited from the outpatient clinic for screening and treatment of late effects of childhood cancer and/or HSCT of the Internal Medicine Department (Leiden University Medical Centre, the Netherlands). Healthy controls were recruited by local advertising in Leiden University, the Netherlands. The control group was in the same age range as the patients and was sex-matched. Laboratory measures in the patient group were performed based on clinical indication and were typically measured within 1 year prior or after CMR examination. No blood samples were drawn in the healthy control group.

### CMR acquisition

The study participants underwent 3 T CMR (Ingenia, Philips Healthcare, Best, the Netherlands), with a dStream Torso anterior coil and a FlexCoverage posterior coil in the table top, resulting in up to 32 coil elements for signal reception. The protocol consisted of standard electrocardiographic (ECG)-triggered two-, three- and four-chamber and short-axis cine balanced steady-state free precession (bSSFP) CMR and ECG-gated gradient-echo 2D velocity-encoded CMR over the mitral valve to quantify LV structure, systolic and diastolic function; 2D velocity-encoded CMR transecting the aortic arch and abdominal aorta to derive aortic stiffness; cardiac native T1 mapping to assess diffuse fibrosis and proton cardiovascular magnetic resonance spectroscopy (^1^H-CMRS) to measure the myocardial triglyceride content. For standardization of the measurement of myocardial triglyceride content, all participants were asked to fast for 6 h and the CMR examinations were scheduled at fixed times (evenings). No CMR contrast material was used.

For the bSSFP cines, typical field-of-view (FOV) was 350 × 350 mm^2^ (long-axis) and 400 × 352 mm^2^ (short-axis), acquired voxel size 2.0 × 1.6 mm^2^ (long-axis) and 1.5 × 1.5 mm^2^ (short-axis), slice thickness 8 mm, echo/repetition time (TE/TR) 1.5/3.0 ms, flip angle 45°, number of phases 30 (long-axis) and 35 (short-axis). For short-axis bSSFP cine, the complete LV was imaged, using 14–16 slices, depending on the size of the LV, with 2 slices per breath-hold. Free-breathing through-plane 2D velocity-encoded (venc 100 cm/s) CMR was acquired perpendicular to the mitral valve, with FOV 350 × 350 mm^2^, acquired voxel size 2.5 × 2.5mm^2^, slice thickness 8 mm, flip angle 10°, TE/TR 2.9/4.6 ms, number of phases 40.

Aortic pulse wave velocity (PWV) was quantified as a measure of aortic stiffness. For aortic PWV, first, a double-oblique sagittal scout view of the aorta was obtained. Then, two free-breathing through-plane 2D velocity-encoded CMR scans were acquired, one transecting the ascending aorta (venc 150 cm/s) and one transecting the abdominal aorta, above the aortic bifurcation (VENC 100 cm/s). Typical imaging parameters were: FOV 350 × 282 mm^2^, slice thickness 8 mm, acquired voxel 2.8 × 2.8 mm^2^, flip angle 20°, TE/TR 2.5/4.4 ms, temporal resolution 10 ms.

Native T1 mapping was acquired in the mid-ventricular short-axis slice, with breath-holding, using the 5s(3s)3s modified Look-Locker inversion recovery (MOLLI) scheme. Typical imaging parameters were: FOV 350 × 300 mm^2^, slice thickness 8 mm, acquired voxel size 2.1 × 2.1 mm^2^, flip angle 20°, TE/TR 1.1/2.3 ms, SENSitivity Encoding (SENSE) factor 2. ^1^H-MRS was performed as described previously [19]. In summary, a voxel of 40 x 15 x 25 mm^3^ was placed in the interventricular septum. For the acquisition with and without water suppression, 48 and 6 signal averages were obtained, respectively. ECG-triggering was used to acquire ^1^H-CMRS at 200 ms after the R-wave, and a respiratory navigator, tracking the lung-liver interface, for acquisition at end-expiration. A high permittivity pad was placed on the chest for improved signal-to-noise ratio.

### Image analysis

CMR data were analyzed using MASS Research Software V2016-EXP (Leiden University Medical Center, the Netherlands) for LV structure and function and aortic PWV, custom-made software for further analysis of the aortic velocity-time curves [20], Medis Suite 3.0 (Medis Medical Imaging systems, Leiden, the Netherlands) for LV systolic and diastolic strain (QStrain 2.0) and native T1 (QMap 2.2.18), and the Java-based magnetic resonance user interface (jMRUI v5.0; MRUI Consortium) for ^1^H-CMRS. The image analysis was blinded to patient or healthy control status.

**Fig. 1:**
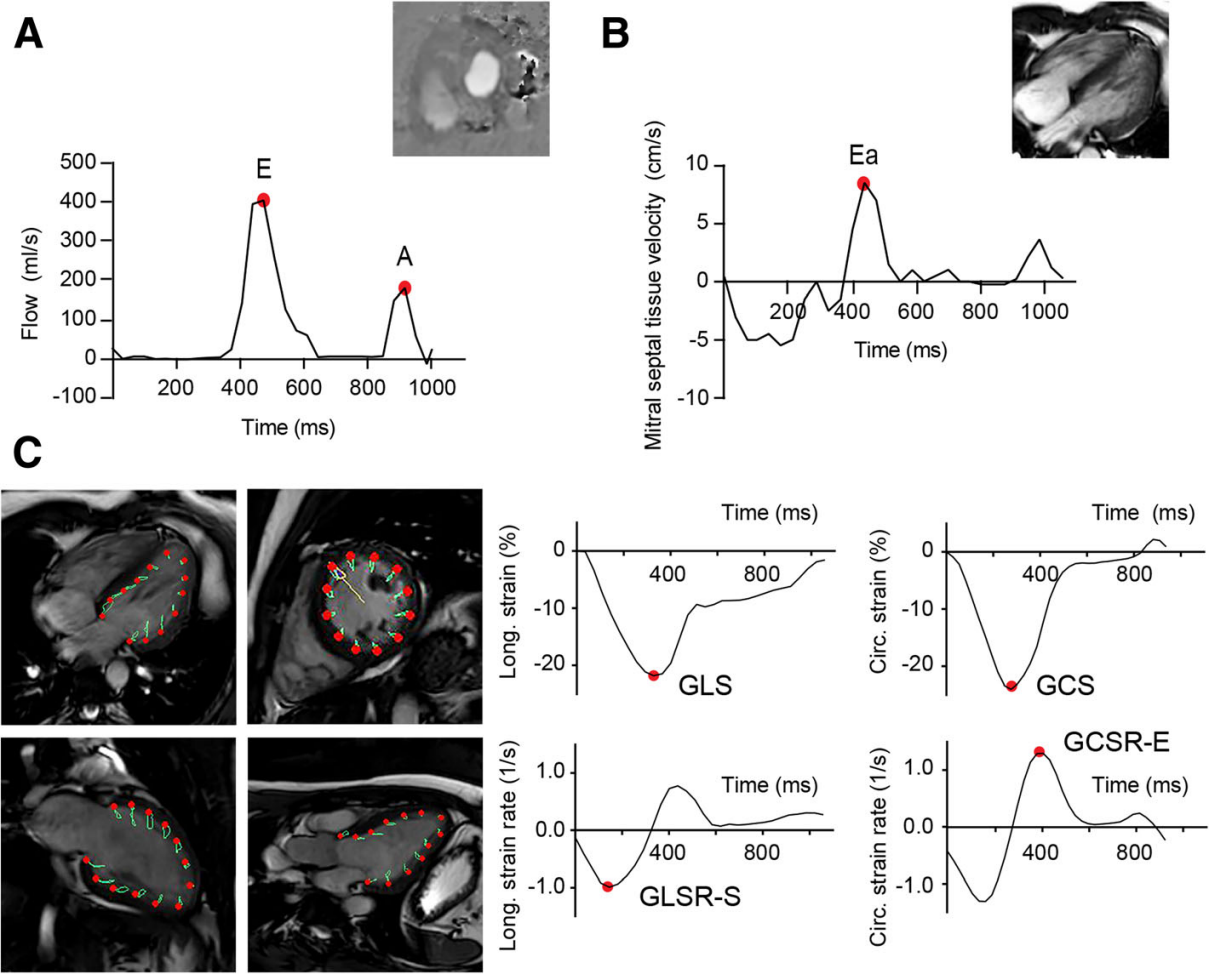
A 22-year-old man, who was transplanted for a non-malignant bone marrow failure disorder at the age of 8 years. **a** the transmitral E/A ratio (early peak filling rate/late peak filling rate) was measured using 2D velocity-encoded CMR (*left panel*) and Ea (early peak diastolic mitral septal tissue velocity) was derived from the four-chamber long-axis relaxation *(right panel)*. LV filling pressure was estimated by the ratio of the transmitral early peak maximum velocity and the early peak diastolic mitral septal tissue velocity. **b** From two, three- and four-chamber and mid-ventricular short-axis cine CMR (*left panel*), the longitudinal and circumferential strain and strain rate curves were extracted (*right panel*). The myocardial features at the endocardial borders (red dots), which were automatically tracked throughout the cardiac cycle (green lines), were manually annotated in the end-diastolic and end-systolic phase. GLS: global longitudinal strain; GCS: global circumferential strain; GLSR-S: global longitudinal peak systolic strain rate; GCSR-E: global circumferential early peak diastolic strain rate

For LV mass and volumes, the endocardial and epicardial LV borders were manually outlined in the end-diastolic and end-systolic phase, with exclusion of the LV papillary muscles. For the feature tracking based strain calculations, the manually annotated endocardial LV borders were automatically tracked throughout the cardiac cycle. Global longitudinal strain (GLS), global longitudinal peak systolic strain rate (GLSR-S) and global longitudinal early peak diastolic strain rate (GLSR-E) were calculated based on the two-, three- and four-chamber cine images. Global circumferential strain (GCS), global circumferential peak systolic strain rate (GCSR-S) and global circumferential early peak diastolic strain rate (GCSR-E) were extracted from the mid-ventricular short-axis cine slice (Fig. 1). The ratio of the transmitral early and late peak filling rate (E/A ratio) and the transmitral early peak maximum velocity were derived from the 2D velocity-encoded scans (Fig. 1), as described previously [21]. The transmitral filling rate was measured after correction for the through-plane background velocity of the LV myocardial wall. Furthermore, the early diastolic mitral septal tissue velocity (Ea) was extracted from the four-chamber cine images. Subsequently, the estimated LV filling pressure (ratio of transmitral early peak maximum velocity without through-plane myocardial motion correction and early diastolic mitral septal tissue velocity) was calculated (E/Ea ratio).

**Fig. 2:**
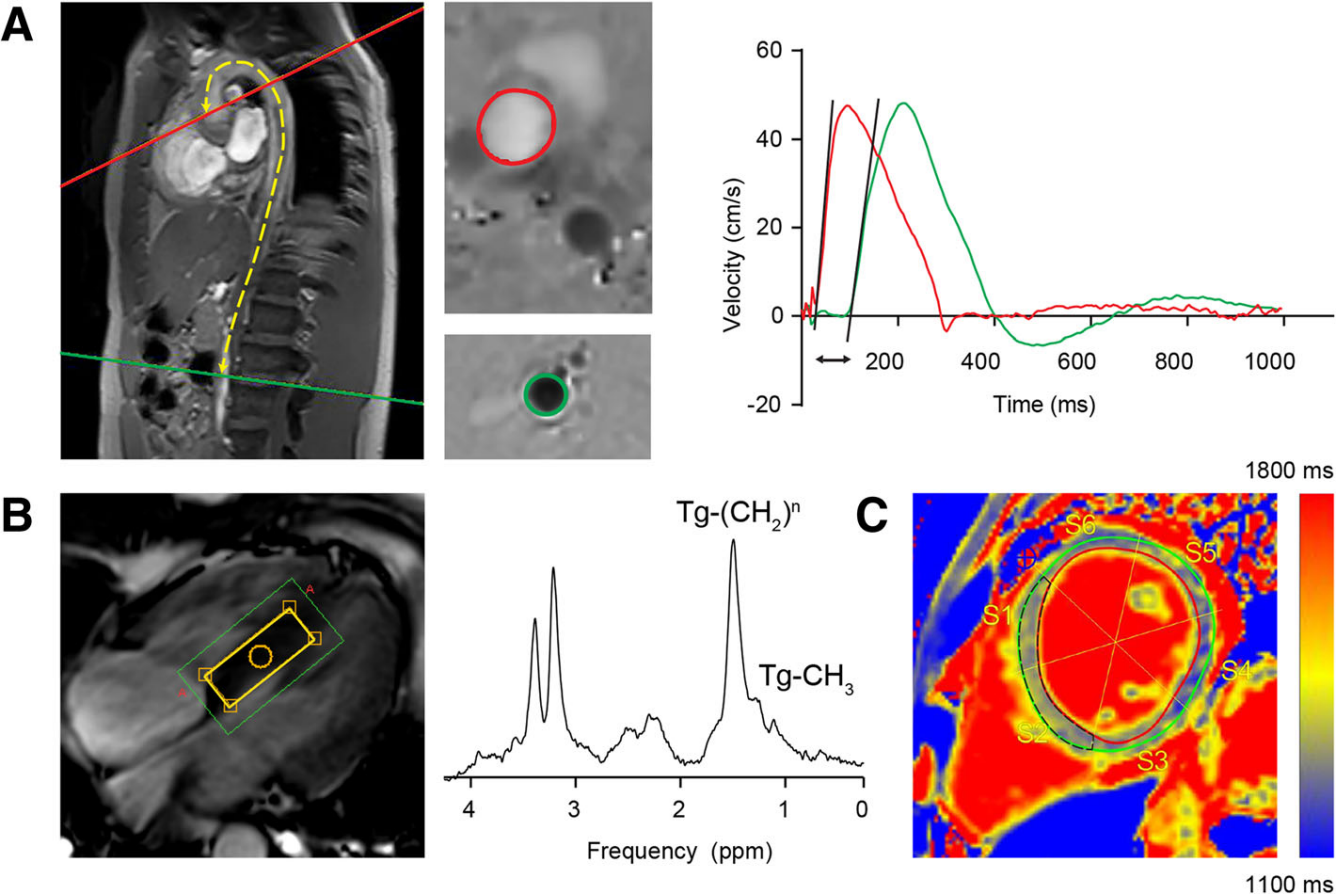
The same patient as in Fig. 1 is presented. **a** Aortic pulse wave velocity was calculated from through-plane 2D velocity-encoded CMR transecting the ascending aorta (red) and the abdominal aorta, above the aortic bifurcation (green) (*left panel*), according to: aortic pulse wave velocity = ∆x/∆t, with ∆x: the distance between the ascending and abdominal aorta (yellow dotted line) and ∆t: transit time of the onset of the systolic velocity wave front (black arrow) (*right panel*). **b** Proton-cardioavascular magnetic resonance spectroscopy (^1^H-CMRS) was used to measure the myocardial triglyceride content. The voxel of interest was placed in the mid-ventricular septum (yellow box) (*left panel*). Myocardial triglyceride content was calculated as Tg-(CH_2_)^n^ and Tg-CH_3_ relative to the sum of the triglyceride and the unsuppressed water signal (not shown). Triglycerides were measured using the water-suppressed spectrum (*right panel*). **c** Native T1 was measured in the mid-ventricular septal segments in short-axis view (black, dotted region of interest)

Aortic PWV was calculated by dividing the distance between ascending and abdominal aorta by the transit time of the onset of the systolic velocity wave front (Fig. 2), as described previously [20]. In short, the aortic path length was measured manually along the aortic centerline on the double-oblique sagittal aorta scout scan. The onset of the systolic wave front was automatically determined from the resulting velocity graph by the intersection point of the constant horizontal diastolic velocity and upslope of the systolic wave front, modeled by linear regression (using the velocity values between 20 and 80% of the total range) along the upslope. T1 maps were constructed after manual in-plane motion correction of the T1 images, using a pixel-wise, mono-exponential three-parameter fit for the T1 relaxation curve. For the measurement of native T1, a region-of-interest in the mid-ventricular septum was drawn (Fig. 2). Myocardial triglyceride content was expressed as the percentage of triglyceride methyl (at 0.9 ppm) and triglyceride methylene (at 1.3 ppm) relative to the sum of the triglyceride signal and the unsuppressed water signal (at 4.7 ppm) (Fig. 2) [22].

### Statistics

Statistical analyses were performed in SPSS 23 (International Business Machines, Armonk, New York, USA). Normal distribution was checked using the Shapiro-Wilk test. Differences between groups were tested for statistical significance using the Student’s t-test or the Mann-Whitney U test for normally and non-normally distributed variables, respectively. Patients with prior HSCT vs. controls were compared and additional analyses were performed for patients with HSCT for malignancies vs. controls. Levels for statistical significance were set at *P* < 0.05 and all tests were two-sided. Additionally, we performed a post-hoc power analysis (alfa 0.05, two-sided).

## Results

In total, sixteen patients (22.1 ± 1.5 years, 11/16 (69%) men) and sixteen healthy controls (22.1 ± 1.8 years, 11/16 (69%) men) were included and analyzed. In one patient, ^1^H-CMRS data were not analyzed due to insufficient quality. Also, in one patient 2D velocity-encoded CMR over the mitral valve was not acquired due to imaging time constraints. Height and body surface area (BSA) in the patient group were lower compared to the healthy control group. The anthropometric characteristics are presented in Table 1.

**Table 1:** Demographic and anthropometric characteristics

	Pediatric HSCT recipients (*n* = 16)	Healthy controls (*n* = 16)	*P* value
Men, *n* (%)	11 (69%)	11 (69%)	1.000
Age, y	22.1 ± 1.5	22.1 ± 1.8	1.000
Length, cm	173 ± 7	179 ± 8	0.017*
Weight, kg	66.6 ± 10.9	72.1 ± 6.9	0.095
Body mass index (BMI), kg/m^2^	22.4 ± 3.6	22.5 ± 2.7	0.929
Body surface area (BSA), m^2^	1.78 ± 0.16	1.89 ± 0.11	0.029*

Values are presented as numbers (percentages) or means ± standard deviations. *P < 0.05. BSA based on the Mosteller formula

In ten patients, HSCT was indicated for a malignant disorder. Seven patients were treated with anthracyclines prior to HSCT, eight received total body irradiation (typically unfractionated 7.5 Gy or 2 × 6 Gy) and fourteen were given high-dose cyclophosphamide (> 1 g/m^2^) for HSCT conditioning. Five patients had a ferritin level above 250 μmol/L. In two of them, cardiac T2* was assessed upon clinical indication. In both, the T2* values were not suggestive of myocardial iron deposition. Clinical and biochemical patients characteristics are presented in Tables 2 and 3.

**Table 2:** Clinical patient characteristics

	Pediatric HSCT recipients (*n* = 16)
Transplant-related characteristics	
Age at time of HSCT, years	7.2 ± 5.3
Time after HSCT, years	14.8 ± 5.0
Malignant disorder, *n* (%)	10 (62.5%)
ALL or AML	8
MDS or CML	2
Non-malignant disorder, *n* (%)	6 (37.5%)
Hematological disease	4
Other	2
Anthracycline therapy, *n* (%)	7 (43.8%)
< 300 mg/m^2^	5
≥ 300 mg/m^2^	2
Radiotherapy, *n* (%)	8 (50.0%)
Total body irradiation	7
Chest irradiation	1
Cyclophosphamide therapy, *n* (%)	14 (87.6%)
< 1 g/m^2^	1
≥ 1 g/m^2^	13
Allogeneic HSCT, *n* (%)	15 (94%)
HLA-identical sibling	6
Other related or unrelated donor	9
Graft versus host disease, *n* (%)	5 (31.3%)
Acute	2
Chronic	3
Clinical parameters	
Systolic blood pressure, mmHg	121 ± 12
Diastolic blood pressure, mmHg	76 ± 9
Pulse, beats per minute	75 ± 12

Values are presented as numbers (percentages) or means ± standard deviations. *HSCT* hematopoietic stem cell transplantation, *ALL* acute lymphatic leukemia, *AML* acute myeloid leukemia, *MDS* myelodysplastic syndrome, *CML* chronic myeloid leukemia

**Table 3:** Biochemical patient characteristics

	Pediatric HSCT recipients (*n* = 16)	Normal values
Fasting glucose, mmol/L	5.1 ± 0.8	3.1–6.4
Total cholesterol, mmol/L	4.90 ± 1.20	3.90–7.30
HDL-cholesterol, mmol/L	1.30 ± 0.32	0.80–2.30
LDL-cholesterol, mmol/L	2.69 ± 0.87	0.00–3.37
Triglycerides, mmol/L	1.87 ± 1.29	0.80–2.30
Alanine aminotransferase (ALT), U/L	Men: 32 ± 17	0–45
	Women: 15 ± 4	0–34
Aspartate aminotransferase (AST), U/L	Men: 31 ± 16	0–35
	Women: 21 ± 2	0–31
Gamma-glutamyl transpeptidase (GGT), U/L	Men: 40 ± 24	0–55
	Women: 19 ± 10	0–38
Free thyroxin (FT4), pmol/L	15.9 ± 2.6	12.0–22.0
Thyroid-stimulating hormone (TSH), mU/L	2.695 ± 1.472	0.300–4.800
eGFR (CKD-EPI) > 60 mL/min/1.73m^2^, n (%)	16 (100%)	NA
Ferritin, μmol/L	Men: 187, 183 (61, 910)	35–260
	Women: 31, 437 (8, 792)	10–150
Ferritin > 250 μmol/L, *n* (%)	5 (31.3%)	NA
Hemoglobin, mmol/L	Men: 9.0 ± 0.7	8.5–11.0
	Women: 8.4 ± 0.9	7.5–10.0

Values are presented as numbers (percentages), mean ± standard deviation or median, interquartile range (minimum, maximum), if the distribution was skewed. Separate values are reported for men (*n* = 11) and women (*n* = 5), if applicable. *HSCT* hematopoietic stem cell transplantation

**Fig. 3:**
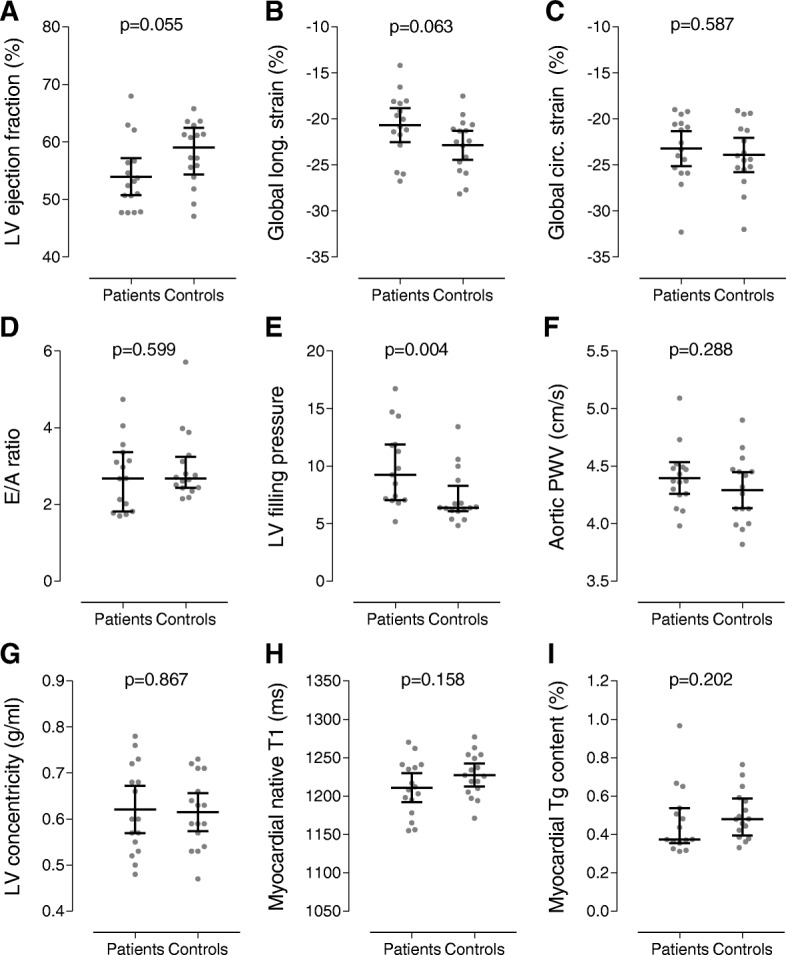
CMR measurements with means and 95% confidence intervals and median and interquartile ranges for normally and non-normally distributed data, respectively. Despite a non-significantly lower left ventricular (LV) systolic function (**a-c**) and lower LV diastolic function (**d-e**) as indicated by the increased estimated LV filling pressure, the CMR parameters for aortic stiffness (**f**) and LV structure (**g**) and myocardial tissue characteristics (**h-i**) were comparable for the patients who received HSCT and the healthy controls. Abbreviations as in Table 4

Patients as compared to the healthy controls had a higher E/Ea ratio (9.92 ± 3.42 vs. 7.24 ± 2.29, *P =* 0.004), while the E/A ratio (2.76 ± 0.92 vs. 2.97 ± 0.91, *P =* 0.599) and diastolic strain rates were comparable. There was a trend towards a lower LV ejection fraction (LVEF) (54 ± 6 vs. 58 ± 5%, *P =* 0.055) and lower GLS (-20.7 ± 3.5 vs. -22.9 ± 3.0%, *P =* 0.063), whereas GCS was preserved (-23.2 ± 3.6 vs. -23.9 ± 3.5%, *P =* 0.587). Stroke volume (83 ± 15 vs. 101 ± 17 mL, *P =* 0.003) but also cardiac output (5.2 ± 1.0 vs. 6.5 ± 1.0 L, *P =* 0.001) and cardiac index (2.9 ± 0.6 vs. 3.4 ± 0.5 L/m^2^, *P =* 0.021) were lower. In contrast, aortic PWV (4.40 ± 0.26 vs. 4.29 ± 0.29 m/s, *P =* 0.288), LV concentricity (0.62 ± 0.10 vs. 0.61 ± 0.08 g/mL, *P =* 0.867), native T1 (1211 ± 36 vs. 1227 ± 28 ms, *P =* 0.158) and myocardial triglyceride content (0.47 ± 0.18 vs. 0.50 ± 0.13%, *P =* 0.202) were comparable. CMR results are presented in Table 4 and Fig. 3.

**Table 4:** CMR parameters

	Pediatric HSCT recipients (*n* = 16)	Healthy controls (*n* = 16)	*P* value
LV mass and dimensions			
Mass, g	95 ± 18	108 ± 23	0.078
Mass/BSA, g/m^2^	53 ± 9	57 ± 10	0.328
End-diastolic volume, mL	155 ± 29	176 ± 30	0.052
End-diastolic volume/BSA, mL/m^2^	87 ± 14	93 ± 13	0.238
Mass/end-diastolic volume, g/mL	0.62 ± 0.10	0.61 ± 0.08	0.867
LV systolic function			
Stroke volume, mL	83 ± 15	101 ± 17	0.003*
Cardiac output, L	5.2 ± 1.0	6.5 ± 1.0	0.001*
Cardiac index, L/m^2^	2.9 ± 0.6	3.4 ± 0.5	0.021*
Ejection fraction, %	54 ± 6	58 ± 5	0.055
Global longitudinal strain, %	−20.7 ± 3.5	−22.9 ± 3.0	0.063
Global circumferential strain, %	−23.2 ± 3.6	−23.9 ± 3.5	0.587
Longitudinal peak systolic strain rate, 1/s	−0.95 ± 0.20	−1.06 ± 0.21	0.137
Circumferential peak systolic strain rate, 1/s	−1.21 ± 0.22	−1.32 ± 0.36	0.216
LV diastolic function			
E/A ratio	2.76 ± 0.92	2.97 ± 0.91	0.599‡
Estimated LV filling pressure	9.92 ± 3.42	7.24 ± 2.29	0.004‡
Longitudinal early diastolic strain rate, 1/s	1.01 ± 0.26	1.15 ± 0.35	0.224
Circumferential early diastolic strain rate, 1/s	1.28 ± 0.29	1.38 ± 0.35	0.396
LV myocardial tissue characteristics			
Myocardial triglyceride content, %	0.47 ± 0.18	0.50 ± 0.13	0.202‡
Native T1 relaxation time, ms	1211 ± 36	1227 ± 28	0.158
Aortic stiffness			
Aortic pulse wave velocity, m/s	4.40 ± 0.26	4.29 ± 0.29	0.288

Values are presented as means ± standard deviations. *P < 0.05. ‡*P* value not based on the Student’s t-test but on the Mann-Whitney U test. *HSCT* hematopoietic stem cell transplantation, *LV* left ventricle, *BSA* body surface area, *E/A ratio* ratio of early and late peak filling rate. Estimated LV filling pressure: ratio of transmitral early peak velocity and early diastolic mitral septal tissue velocity (E/Ea)

When comparing the subgroup with HSCT for malignancies with controls, the higher E/Ea persisted (10.33 ± 3.55 vs. 7.24 ± 2.29, *P =* 0.013). Additionally, LVEF (52 ± 4 vs. 58 ± 5%, *P =* 0.008), GLS (− 19.8 ± 3.3 vs. -22.9 ± 3.0%, *P =* 0.022) and GCSR-S (-1.13 ± 0.15 vs. -1.32 ± 0.26 1/s, *P =* 0.047) were lower. For the HSCT recipients treated for malignancies, aortic PWV was: 4.39 ± 0.18 m/s (*P =* 0.36 vs. controls), LV concentricity: 0.63 ± 0.10 g/mL (*P =* 0.73), native T1: 1208 ± 36 ms (*P =* 0.138) and myocardial triglyceride content: 0.53 ± 0.21 (*P =* 0.69).

### Post-hoc power analysis

We had 0.90 power to detect a statistically significant difference if the HSCT population would have had an aortic PWV of 4.64 m/s or higher (+ 0.33 m/s higher as compared to the healthy controls), myocardial triglyceride content of 0.69% or higher (+ 0.19%) and native T1 of 1268 ms or higher (+ 39 ms). These differences in aortic PWV, myocardial triglyceride content and native T1 are comparable to approximately 3, 20 and 35 years of normal aging, respectively [23–25].

## Discussion

Our study showed that young adults with a history of pediatric HSCT, as compared to healthy controls in the same age range, have subclinical impairments in LV diastolic function and tend to have a lower LV systolic function, whereas aortic stiffness and myocardial tissue characteristics are comparable. Our results indicate that CMR-derived LV diastolic parameters, rather than aortic PWV, cardiac native T1 or myocardial triglyceride content, may be early markers of cardiovascular deterioration after HSCT.

### LV function

The estimated LV filling pressure was found to be increased after childhood HSCT, which is indicative of reduced LV diastolic function. When LV myocardial relaxation progressively deteriorates, LV filling pressure becomes increased to compensate for the impaired LV diastolic filling [26]. Accordingly, in the patients who received HSCT during childhood, the E/A ratio was comparable as in the controls, whereas LV filling pressure was higher. In contrast to the impairments in LV diastolic function, the decrease in LV systolic function in the HSCT group compared to the control group was non-significant. This observation is in keeping with prior longitudinal studies using echocardiography, which showed that diastolic dysfunction precedes systolic dysfunction in response to cardiotoxic exposures [27, 28]. Anthracycline-related cardiomyopathy is considered to result from free radical formation, the consequent mitochondrial dysfunction and myofibrillar disarray, and eventual myocyte necrosis; in turn, with the ongoing remodeling of the injured heart, functional impairments may progressively develop [2]. Furthermore, irradiation may cause microcirculatory damage, leading to myocardial ischemia and cell death [2]. However, despite the reduced LV diastolic function, there was no interstitial fibrosis based on native T1 mapping. Furthermore, aortic stiffening or myocardial steatosis did not appear contributing factors to the abnormalities in LV diastolic function. Hence, when using CMR, LV diastolic parameters in particular may be important in the monitoring of HSCT-related cardiovascular deteriorations.

In our study, the trend for lower LVEF and significantly lower cardiac index in the pediatric HSCT recipients compared to the controls seemed to be related to impairments in GLS rather than GCS. GLS and GCS, respectively, can be interpreted as measures of the contractility of the cardiomyocytes in oblique orientation in the subendocardium and those in circumferential arrangement in the mesomyocardium [29, 30]. Accordingly, our results may suggest that the HSCT-related therapies predominately affect the subendocardial myocardium, and not the midwall. In the subgroup with a history of a malignant disorder, longitudinal and circumferential systolic strain-derived parameters were lower as compared to healthy controls; this is consistent with previous studies which assessed LV systolic strain parameters in patients receiving anthracyclines [31, 32].

We hypothesized that aortic stiffening or altered myocardial tissue characteristics would be early markers of cardiovascular disturbances after HSCT, preceding manifest LV dysfunction. Of interest, as chemotherapy is considered to disrupt mitochondrial function and irradiation has been demonstrated to induce microcirculatory damage [2], several other CMR-derived parameters may have potential for the early detection of cardiac effects of HSCT, for example phosphorus CMRS (^31^P-CMRS) for the assessment of myocardial energetics and perfusion imaging [33].

### Aortic stiffness

No differences were observed in aortic stiffness between the HSCT study population and the healthy controls. Based on previous studies, we expected to find a significantly higher aortic stiffness, at least in the patients with HSCT for a malignant disorder, because of the pre-transplant cardiotoxic exposures. Anthracycline and/or chest irradiation in the treatment of the primary cancer may cause endothelial damage due to the generation of reactive oxygen species [34, 35]. Also, the conditioning in allogeneic HSCT, including total body irradiation and/or chemotherapy, is considered to initiate disruption of the endothelium [36]. Subsequently, the structural changes in the vascular matrix and the disturbed endothelial function may increase the vascular tone of the arterial wall [2]. In addition, metabolic disturbances caused by the immune dysregulation and iron overload are well recognized adverse effects of HSCT [12, 37]. There is extensive evidence for an elevated aortic stiffness due to hypertension, dyslipidemia and hyperglycemia in the general population [14]. The role of iron overload in endothelial dysfunction is less evident, although iron chelation in coronary artery disease has been shown to improve endothelium-dependent vasodilation [38].

Previous longitudinal studies in patients with breast cancer, lymphoma or leukemia showed that aortic PWV increases by approximately 1.5 to 2-fold upon anthracyclines in the first 4 to 6 months, also for low or moderate dosages, compared to the pre-treatment measurements [39, 40]. Another study measured a higher carotid artery stiffness in children who had received allogeneic HSCT, for malignant or non-malignant disease, even though this study population showed only minor, subclinical metabolic derangements, and no other cardiovascular impairments were detected [41]. However, the increased aortic stiffness due to cardiotoxic exposures may decrease after the cardiotoxic therapy has been discontinued, as was shown in breast cancer patients who were followed before and 1, 4 and 14 months after anthracycline or trastuzumab chemotherapy [42]. We measured aortic PWV after a mean time of 14.8 ± 5.0 years after HSCT. Possibly, there may have been changes in aortic PWV acutely after pediatric HSCT, which may have normalized after several years.

### Myocardial tissue characteristics

There were no differences in myocardial tissue characteristics and LV concentricity between the HSCT study population and the healthy controls, which otherwise would have been indicative of LV remodeling. To our knowledge, this is the first study in which native T1, as a measure of interstitial fibrosis, and myocardial triglyceride content, as a measure of metabolic remodeling, have been assessed in young adults with prior HSCT. It should be noted that diffuse fibrosis can be estimated based on calculating native T1 or the extracellular volume fraction (ECV); the latter requires CMR contrast administration [17]. Recent CMR studies have demonstrated that both native T1 and ECV are increased in middle-aged patients with prior anthracycline therapy [43, 44]. Other studies in adolescents exposed to anthracyclines, however, reported native T1 and/or ECV values within the normal range [45, 46]; nonetheless, a correlation was found between the cumulative anthracycline dose and native T1 and ECV [46]. In contrast to these observations suggestive of diffuse fibrosis, focal fibrosis, visualized with late gadolinium enhancement CMR, is uncommon after anthracycline exposure, even when systolic function is subnormal [44–47]. In our HSCT study population as compared to the controls, we did not find a higher native T1, even though a lower LVEF was measured. Also, when analyzing the patients who received HSCT for a malignant indication separately, no difference with the control group was found. Therefore, in contrast to middle-aged patients with prior anthracycline therapy as described in other studies, we may conclude that there is no myocardial fibrosis, at least not substantial, in young adults with a history of childhood HSCT as compared to healthy controls.

From previous studies it is known that the metabolic syndrome, presumably due to changes in myocardial fatty acid uptake and/or oxidation, is associated with myocardial steatosis [18, 48]. Although allografting increases the risk of early development of the metabolic syndrome [12], our HSCT study population aged 18–25 years had on average no obesity, impaired fasting glucose or hypertriglyceridemia. Possibly, myocardial steatosis may arise after HSCT at middle-age, when metabolic disorders may have developed, while myocardial triglyceride content remains within the normal ranges at younger age. Based on our findings, we may conclude that myocardial triglyceride content does not represent an early marker of cardiometabolic disease after pediatric HSCT in the young adult population.

### Strengths

Strength of this study is the comprehensive CMR evaluation, including measures of both LV systolic and diastolic function, vascular function and myocardial tissue characteristics. CMR rather than ultrasound is the most accurate non-invasive modality to quantify aortic stiffness [49]. Diffuse fibrosis was measured using native T1 mapping, which is a non-contrast technique; contrast-enhanced CMR can be considered less suitable for screening programs. Cardiac ^1^H-CMRS is the gold standard for non-invasive assessment of myocardial steatosis [48]. In clinical practice, echocardiography is commonly used for follow-up after cardiotoxic therapies [11]. Compared to echocardiography, little is known regarding the value of CMR for cardiovascular screening after HSCT.

### Limitations

Our study did not comprise CMR before and shortly after HSCT, but had a cross-sectional design because of ethical considerations. Therefore, we cannot exclude temporary deteriorations in, for example, aortic stiffness. Our study population was too small to evaluate the patient group with HSCT for non-malignant disease separately. The observed abnormalities in LV diastolic function and the non-significantly lower LV systolic function seemed to be driven by the patients who had received HSCT for a malignant disease. However, based on our study we cannot rule out late LV functional impairment after HSCT for non-malignant disease. Also, we were not able to assess the correlations between the different HSCT conditioning regimens, graft-versus-host-disease or iron overload and cardiovascular measures. However, this study was aimed at identifying CMR parameters which may show deteriorations before overt cardiovascular disease develops, and not to identify HSCT-related factors which may be helpful in late cardiovascular disease risk stratification. Echocardiography was not applied in the present study, which would have allowed for comparison of CMR with echocardiography for the early detection of cardiac effects of HSCT.

As a measure of interstitial fibrosis, ECV may be preferable. The native T1 value may be affected by several processes other than edema or increased myocardial collagen in the extracellular compartment [17]. Hence, based on the native T1 measurements, we may not be able to exclude the presence of diffuse fibrosis in post-HSCT patients. However, as all patients were clinically monitored for cardiac iron deposition and as myocardial steatosis was ruled out based on the ^1^H-MRS measurements, it is unlikely that fibrosis related increases in native T1 might have been cancelled out by myocardial iron or fat related native T1 decreases.

## Conclusion

In young adults who received HSCT during childhood, LV diastolic function was decreased (higher estimated LV filling pressure) and LV systolic function (LVEF and GLS) tended to be reduced as compared to healthy controls, whereas no concomitant differences were found in aortic stiffness (aortic PWV) and myocardial tissue characteristics (native T1 and myocardial triglyceride content). Therefore, when using CMR, the assessment of LV diastolic function in particular is important for early detection of patients at risk of HSCT-related cardiovascular disease. Further research, including longitudinal CMR measurements, is needed to show the predictive value of subclinical LV functional abnormalities for the development of symptomatic cardiovascular disease after HSCT. Also, comparative studies of imaging modalities should reveal whether CMR-derived LV function has additive value for screening programs when performed next to the current echocardiography-based follow-up of the cardiovascular late effects of HSCT. For example, CMR may be used for the selection of patients who require frequent follow-up by standard echocardiography.

## Abbreviations

^1^H-CMRS: Proton cardiovascular magnetic resonance spectroscopy
BMI: Body mass index
BSA: Body surface area
bSSFP: Balanced steady-state free precession
CMR: Cardiovascular magnetic resonance
E/A ratio: Ratio of the transmitral early and late peak filling rate
E/Ea: Estimated left ventricular filling pressure
Ea: Early diastolic mitral septal tissue velocity
ECG: Electrocardiogram
ECV: Extracellular volume
EF: Ejection fraction
FOV: Field-of-view
GCS: Global circumferential strain
GCSR-E: Global circumferential early peak diastolic strain rate
GCSR-S: Global circumferential peak systolic strain rate
GLS: Global longitudinal strain
GLSR-E: Global longitudinal early peak diastolic strain rate
GLSR-S: Global longitudinal peak systolic strain rate
HSCT: Hematopoietic stem cell transplantation
LV: Left ventricle/left ventricular
LVEF: Left ventricular ejection fraction
MOLLI: Modified Look-Locker inversion recovery
PWV: Pulse wave velocity
SENSE: SENSitivity Encoding
TE: Echo time
TR: Repetition time
VENC: Velocity-encoding
